# Betulinic Acid Inhibits Endometriosis Through Suppression of Estrogen Receptor β Signaling Pathway

**DOI:** 10.3389/fendo.2020.604648

**Published:** 2020-12-10

**Authors:** Dongfang Xiang, Min Zhao, Xiaofan Cai, Yongxia Wang, Lei Zhang, Helen Yao, Min Liu, Huan Yang, Mingtao Xu, Huilin Li, Huijuan Peng, Min Wang, Xuefang Liang, Ling Li, Paul Yao

**Affiliations:** ^1^ The Second Affiliated Hospital of Guangzhou University of Chinese Medicine, Guangzhou, China; ^2^ Wuhan Hospital of Traditional Chinese Medicine, Wuhan, China; ^3^ Hainan Maternal and Child Health Hospital, Haikou, China

**Keywords:** betulinic acid, endometriosis, estrogen receptor β (ERβ), inflammation, mitochondria

## Abstract

Endometriosis is an inflammatory gynecological disorder characterized by endometrial tissue growth located outside of the uterine cavity in addition to chronic pelvic pain and infertility. In this study, we aim to develop a potential therapeutic treatment based on the pathogenesis and mechanism of Endometriosis. Our preliminary data showed that the expression of estrogen receptor β (ERβ) was significantly increased, while ERα was significantly decreased, in endometriotic cells compared to normal endometrial cells. Further investigation showed that betulinic acid (BA) treatment suppressed ERβ expression through epigenetic modification on the ERβ promoter, while had no effect on ERα expression. In addition, BA treatment suppresses ERβ target genes, including superoxide dismutase 2 (SOD2), nuclear respiratory factor-1 (NRF1), cyclooxygenase 2 (COX2), and matrix metalloproteinase-1 (MMP1), subsequently increasing oxidative stress, triggering mitochondrial dysfunction, decreasing elevated proinflammatory cytokines, and eventually suppressing endometriotic cell proliferation, mimicking the effect of ERβ knockdown. On the other hand, gain of ERβ by lentivirus infection in normal endometrial cells resulted in increased cell proliferation and proinflammatory cytokine release, while BA treatment diminished this effect through ERβ suppression with subsequent oxidative stress and apoptosis. Our results indicate that ERβ may be a major driving force for the development of endometriosis, while BA inhibits Endometriosis through specific suppression of the ERβ signaling pathway. This study provides a novel therapeutic strategy for endometriosis treatment through BA-mediated ERβ suppression.

## Introduction

Endometriosis (EMS) is a common inflammatory gynecological disease characterized by the presence of endometrial-like lesions located outside the uterus, causing chronic pelvic pain and infertility (1), and affects approximately 5–10% of women during their reproductive ages (2). Diagnosis for EMS is mostly dependent on surgical visualization. Retrograde menstruation is considered to be an important mechanism, while other factors, including endocrine and metabolic pathway, altered immunity, inflammatory response, and metaplasia of the coelom, may also be involved (2). Current treatment for EMS primarily focuses on alleviating symptoms, such as through pain relief, surgical removal, and hormone suppression (3), while development of novel targeted treatment for EMS based on a deeper understanding of its pathological mechanism is still an urgent requirement.

Endometriosis is estrogen dependent, and the expression of estrogen receptor (ER)β in EMS is significantly higher than those in endometrial tissues. High levels of ERβ suppress ERα, resulting in very high ratios of ERβ/ERα (4, 5). Recently, it has been reported that ERβ plays an important role in the pathogenesis of EMS by modulating apoptosis and inflammasome (6). Additionally, ERβ modulates mitochondrial function through nuclear respiratory factor-1 (NRF1) (7) and regulates the basal expression of superoxide dismutase 2 (SOD2) to minimize oxidative stress (8–10). In addition, ERβ modulates the expression of cyclooxygenase-2 (COX2) (1, 4, 11, 12) for inflammation (13) and regulates the expression of matrix metalloproteinases 1 (MMP1) for tissue degradation, cell invasion, and growth (14–16). It has been reported that ERβ plays an important role in EMS pathogenesis by modulating its target genes (6), including NRF1, SOD2, COX2, and MMP1, and specific ERβ suppression, such as ERβ antagonist (17), has been used for treatment of endometriosis, but with many of limitations and side effects. In this case, development of a non-toxic and specific ERβ suppression agent for treatment of endometriosis is still quite necessary.

Betulinic acid (BA) is identified as a pentacyclic triterpene that can be isolated from many natural plants and has been characterized to possess several biological properties, including its ability to inhibit tumor growth (18, 19) through apoptosis (20) and suppress leukemia (21, 22) through modulation of mitochondrial function and oxidative stress (23) while also having an antiviral effect in inhibiting the HIV (18, 24), EBV (25), and HBV viruses (23). Recently, it has been reported that BA suppresses the estrogen signaling pathway either directly or indirectly (26–28), while the detailed mechanism remains largely unknown (29). Here, we hypothesize that BA inhibits endometriosis through ERβ suppression.

In an effort to study the potential mechanism and effect of BA on endometriosis, we evaluated the related gene expression in different cells, finding that ERβ expression was significantly increased in endometriotic cells compared to endometrial cells and BA treatment significantly inhibited the expression of ERβ and its target genes. Further investigation showed that BA suppresses ERβ expression through epigenetic changes on the ERβ promoter. Also, BA treatment significantly increased oxidative stress, induced mitochondrial dysfunction, normalized elevated levels of pro-inflammatory cytokines, and subsequently suppressed cell proliferation and growth in endometriotic cells, mimicking the effect of ERβ knockdown. We conclude that BA inhibits endometriosis by suppression of the ERβ signaling pathway, providing a novel strategy for potential treatment of endometriosis.

## Materials and Methods

A detailed description can be found in Supplementary Information (see **Data S1**), and the primers used in this study are shown in **Table 1**.

**Table 1:** Sequences of primers for the real-time quantitative PCR (qPCR).

Gene	Species	Analysis	Forward primer (5’→3’)	Reverse primer (5’→3’)
β-actin	Human	mRNA	gatgcagaaggagatcactgc	atactcctgcttgctgatcca
ERβ	Human	mRNA	atgatgatgtccctgaccaag	acatcagccccatcattaaca
COX2	Human	mRNA	gcctgacacctttcaaattca	gaacattcctaccaccagcaa
MMP1	Human	mRNA	ttcccagcgactctagaaaca	ttcctgcatttgcttcaattt
NRF1	Human	mRNA	cgaggacacctcttacgatga	tcaaatacatgaggccgtttc
SOD2	Human	mRNA	gcctacgtgaacaacctgaac	tgaggtttgtccagaaaatgc
IL-1β	Human	mRNA	tgggataacgaggcttatgtg	gaacaccacttgttgctccat
IL-6	Human	mRNA	tccaaagatggctgaaaaaga	gctctggcttgttcctcacta
TNF-α	Human	mRNA	tagcccatgttgtagcaaacc	aggacctgggagtagatgagg
PGE2	Human	mRNA	catcagttgagcactgcaaga	tctggcaaaactttcgaagaa
ERβ	Human	ChIP	ctcacattcccactcctctga	gaaacacagaagatattgccaag

### Materials and Reagents

Antibodies for β-actin (sc-47778), COX2 (sc-19999), MMP1 (sc-21731), NRF1 (sc-101102), and SOD2 (sc-30080) were obtained from Santa Cruz Biotechnology. Antibodies for acetyl-histone H4 K5, K8, K12, and K16 (H4K5,8,12,16ac, #PA5-40084) were obtained from Invitrogen. Antibodies for anti-histone H3 acetyl K9, K14, K18, K23, K27 (H3K9, 14, 18, 23, 27ac, ab47915), ERα (ab3575), ERβ (ab3576), H4K20me1 (ab9051), H4K20me3 (ab9053), H4R3me1 (ab17339), H3K9me2 (ab1220), H3K9me3 (ab8898), H3K27me2 (ab24684) and H3K27me3 (ab6002), H2AX (ab20669), and γH2AX (ab2893) were obtained from Abcam. The antibody for 8-oxo-dG (4354-MC-050) was obtained from Novus Biologicals. The 3-nitrotyrosine (3-NT) was measured using 3-Nitrotyrosine ELISA Kit (ab116691 from Abcam) per manufacturers’ instructions. The mitochondrial fraction was isolated using a Pierce Mitochondria Isolation Kit (Pierce Biotechnology) according to manufacturers’ instructions. The protein concentration was measured using the Coomassie Protein Assay Kit (Pierce Biotechnology) per manufacturers’ instructions. Luciferase activity assay was carried out using the Dual-Luciferase™ Assay System (Promega) and the transfection efficiency was normalized using a cotransfected renilla plasmid (23). 17β-estradiol (E2, #E2758) and TNFα (#T0157) were obtained from Sigma.

### Human Cell Lines

Human Endometrial Epithelial Cells HEEC (#ABC-TC4601) and Immortalized Human Endometriotic Epithelial Cell Line 12Z (#ABI-TC278D) were obtained from ACCEGEN Biotechnology. The human primary endometrial epithelial cells (EM) and primary endometriotic epithelial cells (EMT) were a kind gift from Dr. Haimou Zhang (from Hubei University) (30). In some of the experiments, the cells for HEEC, EM, and EMT were conditionally immortalized using a hTERT lentivirus vector with an extended life span to achieve higher transfection efficiency and experimental stability (10, 31). The cells were maintained in DMEM/F12 medium supplemented with 10% FBS, 1% penicillin and streptomycin, and 1% sodium pyruvate at 37°C with 5% CO2.

### Construction of Human Reporter Plasmid

In order to construct ERβ reporter plasmids, the gene promoters (2kb upstream of the transcription start site plus first exon) were amplified from Ensembl ID: ESR2-201 ENST00000267525.10 by PCR from human genomic DNA and subcloned into the pGL3-basic vector (#E1751, Promega) using restriction sites of Mlu I and Hind III with the following primers: ERβ forward: 5’-gcgc-acgcgt- att tca aga cga gcc tgg cca -3’ (Mlu I) and ERβ reverse: 5’- gtac- aagctt- ctg ttt aca ggt aag gtg tgt -3’ (Hind III). To map promoter activity, the related deletion promoter constructs were generated by PCR methods and subcloned into the pGL3-basic vector (23).

### Generation of Human ERβ Expression Lentivirus

The human cDNA for ERβ was obtained from Open Biosystems. The cDNA for human ERβ was subcloned into the pLVX-Puro vector (from Clontech) with the restriction sites of Xho1 and Xba1 using the below primers: ERβ forward primer: 5’- gtac - ctcgag- atg gat ata aaa aac tca cca -3’ (Xho1) and ERβ reverse primer: 5’- gtac - tctaga- tca ctg ctc cat cgt tgc ttc -3’ (Xba1). The ERβ or empty control (CTL) was expressed through Lenti-X™ Lentiviral Expression Systems (from Clontech) per manufacturers’ instructions (32).

### Preparation of ERβ Knockdown (shERβ)

The shRNA lentivirus plasmids for human ERβ (sc-35325-SH) or non-target control (sc-108060) were purchased from Santa Cruz Biotechnology. The related lentiviruses for ERβ and empty control (CTL) were expressed through Lenti-X™ Lentiviral Expression Systems (from Clontech) per manufacturers’ instructions. The purified and condensed lentiviruses were used for *in vitro* gene knockdown. The knockdown efficiency was confirmed by more than 65% of mRNA reduction compared to the control group in cells using real time PCR (see **Table 1**).

### Analysis of Cytokines

Human cytokines, including IL-1β, IL-6, and TNF-α from *in vitro* cell culture supernatant, were measured using Human IL-1 beta/IL-1F2 Quantikine ELISA Kit (#DLB50), Human IL-6 Quantikine ELISA Kit (#D6050), and Human TNF-alpha Quantikine ELISA Kit (#DTA00D), respectively; and the PGE2 release was measured by Prostaglandin E2 Parameter Assay Kit (#KGE004B) according to manufacturers’ instructions from R&D Systems (33).

### Immunostaining

The treated cells were transferred to cover slips, and the cells were fixed in 4% paraformaldehyde for 20 min before being incubated with 0.3% Triton X-100 in PBS for 15 min. After blocking with 5% goat serum in PBS at room temperature for 30 min, cells were incubated with anti-mouse antibody for either 8-oxo-dG (# 4354-MC-050, from Novus Biologicals) or Ki-67 (# sc-101861, from Santa Cruz Biotechnology) for 12 h at 4°C and subsequently with secondary antibody Alexa Fluor 488. The cover slips were then mounted by antifade Mountant with DAPI (staining nuclei, in blue). The photographs were taken using a Confocal Laser Microscope (Leica, 20× lens) and quantitated by Image J. software (34).

**Statistical Analysis**. The data was given as mean ± SEM, and all the experiments were performed at least in quadruplicate unless otherwise indicated. The unpaired Student’s t-tests or one-way analysis of variance (ANOVA) followed by the Turkey−Kramer test was used to determine statistical significance of different groups by SPSS 22 software, and a *P* value of <0.05 was considered significant (35, 36).

## Results

### Expression of ERβ and Its Target Genes Is Significantly Increased, While Expression of ERα Decreased, in Endometriotic Cells Compared to Endometrial Cells

**Figure 1:**
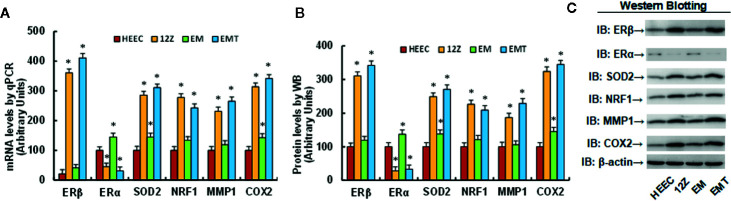
Expression of ERβ and its target genes is significantly increased in endometriotic cells compared to endometrial cells. Human Endometrial Epithelial Cells HEEC, immortalized Human Endometriotic Epithelial Cell Line 12Z, human primary endometrial epithelial cells (EM), and primary endometriotic epithelial cells (EMT) were used for gene expression analysis. **(A)** mRNA levels for ERβ/ERα and its target genes by qPCR, n = 4. **(B)** Quantitation of protein levels, n = 5. **(C)** Representative western blotting pictures for **(B)**. *P < 0.05, *vs* CTL group. Data were expressed as mean ± SEM.

Different cells, including Human Endometrial Epithelial Cells (HEEC), immortalized Human Endometriotic Epithelial Cell Line 12Z, human primary endometrial epithelial cells (EM), and primary endometriotic epithelial cells (EMT), were used for gene expression of ERβ, ERα and its target genes, including SOD2, NRF1, MMP1, and COX2. The results showed that 12Z and EMT cells have ERβ mRNA levels that are 17.2 and 19.6 times higher of than HEEC cells, respectively; On the other hand, ERα mRNA levels were deceased to 45 and 21% in endometriotic 12Z and EMT cells, respectively, compared to its related endometrial HEEC and EM cells. In addition, SOD2 mRNA levels in 12Z and EMT cells increased to 286 and 216%, respectively; NRF1 mRNA levels increased to 244 and 198%, respectively; MMP1 mRNA levels increased to 226 and 187%, respectively; and COX2 mRNA levels increased to 314 and 211%, respectively, compared to HEEC cells. Furthermore, we found that there was no difference in mRNA levels of ERβ, NRF1, and MMP1 in EM cells, but levels were 1.45 and 1.43 times higher, respectively, then in HEEC cells (see **Figure 1A**). In addition, we measured the protein levels for those genes and found that ERβ levels in 12Z and EMT cells were 3.11 and 3.42 times higher than that of HEEC cells, respectively, and an expression pattern similar to that of the mRNA was observed for genes of SOD2, NRF1, MMP1, and COX2 (see **Figures 1B, C**, and **S1**). We conclude that expression of ERβ and its target genes is significantly increased, while expression of ERα decreased, in endometriotic cells compared to endometrial cells.

### Betulinic Acid Suppresses the ERβ Signaling Pathway by Modulation of Epigenetic Changes on the ERβ Promoter and Subsequent Expression

**Figure 2:**
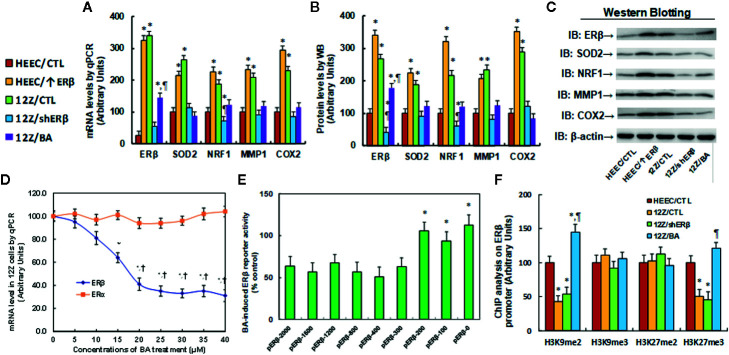
Betulinic acid suppresses the ERβ signaling pathway by modulation of epigenetic changes on the ERβ promoter and subsequent expression. **(A–C)** The conditional immortalized HEEC or 12Z cells were either treated by 20 µM betulinic acid (BA) for 24 h or infected by empty control (CTL), ERβ expression (↑ERβ), or ERβ knockdown (shERβ) lentivirus. The cells were then harvested for gene expression analysis. **(A)** The mRNA levels qPCR, n = 4. **(B)** Quantitation of protein levels, n = 5. **(C)** Representative western blotting pictures for **(B)**. *P < 0.05, *vs* HEEC/CTL group; ^¶^ *P* < 0.05, *vs* 12Z/CTL. **(D)** 12Z cells were treated by different concentrations of betulinic acid for 24 h, and the cells were harvested for analysis of ERα and ERβ mRNA by qPCR, n = 4. **(E)** 12Z cells were transiently transfected by either ERβ full length (pERβ-2000) or deletion reporter plasmids. After 24 h, the cells were treated by either control (CTL) or 20 µM BA for 24 h, and BA-induced relative ERβ reporter activities (% control) were calculated, n = 5. *P < 0.05, *vs* pERβ-2000 group. **(F)** Treated cells were harvested to measure epigenetic changes on the ERβ promoter by ChIP analysis, n = 4. *P < 0.05, *vs* HEEC/CTL group. Data were expressed as mean ± SEM.

We first evaluated the potential effect of betulinic acid (BA) on the gene expression of ERβ and its target genes. The conditional immortalized HEEC or 12Z cells were either treated with 20 µM betulinic acid (BA) for 24 h or infected by a lentivirus of the empty control (CTL), ERβ expression (↑ERβ), or ERβ knockdown (shERβ) groups, before being harvested for gene expression analysis. The results showed that HEEC/↑ERβ and 12Z/CTL treatments result in ERβ mRNA levels that are 13.0 and 13.6 times higher than that of HEEC cells. Additionally, HEEC/↑ERβ and 12Z/CTL treatments have increased SOD2 mRNA levels to 215 and 264%, respectively; increased NRF1 mRNA levels to 226 and 187%, respectively; increased MMP1 mRNA levels to 234 and 208%, respectively; and increased COX2 mRNA levels to 295 and 229%, respectively, compared to the HEEC/CTL group. This indicates that a gain of ERβ by lentivirus infection in endometrial cells (HEEC/↑ERβ) increases expression of ERβ and its target genes, mimicking the effect of endometriotic cells, such as the 12Z/CTL group. On the other hand, ER knockdown in 12Z cells (12Z/shERβ) decreased the expression of ERβ and its target genes to levels that were similar to that of the HEEC/CTL group, and interestingly, BA treatment (12Z/BA) mimicked the effect of shERβ and normalized endometriosis (12Z cells)-mediated increased expression of ERβ and its target genes (see **Figure 2A**). We also measured the protein levels for those genes and found that HEEC/↑ERβ and 12Z/CTL treatments resulted in ERβ levels that were 3.41 and 2.68 times higher than that of HEEC cells, respectively, and an expression pattern similar to that of the mRNA was observed for the SOD2, NRF1, MMP1, and COX2 genes (see **Figures 2B, C**, and **S2**). These results indicate that BA can suppress gene expression of ERβ and its target genes. We then measured the potential effect of BA on ERβ/ERα expression using dose-response curve in endometriotic 12Z cells, which showed that 20 µM of BA achieved the maximum inhibition effect on ERβ mRNA expression after a 24-h treatment, while BA treatment showed no effect on ERα expression (see **Figure 2D**). We also investigated the potential effect in endometrial HEEC cells, the results showed that 20 µM of BA had no significant effect on the expression of ERα and ERβ, while when the dose of BA reached to 30 μM, it increased ERα, but decreased ERβ expression very slightly (see **Figure S3**), indicating that BA treatment can specifically suppress ERβ expression in endometriotic cells. Afterwards, we investigated the potential molecular mechanism for betulinic acid-mediated ERβ suppression. In order to identify the betulinic acid responsive element on the ERβ promoter, a series of progressive 5’-promoter deletion constructs for the ERβ promoter were generated by PCR methods, including -2000, -1600, -1200, -800, -400, -300, -200, -100, and -0 deletion constructs (numbered according to Ensembl gene ID: ESR2-201 ENST00000267525.10, and the transcription start site was marked as 0). All of these constructs were transfected into 12Z cells for the analysis of ERβ reporter activity in the presence of either control (CTL) or 20 µM betulinic acid for 24 h, and the betulinic acid-induced relative reporter activities (% control) were calculated. Our results showed that BA-induced relative reporter activities were around 60% compared to the control group for the deletion constructs of -2000, -1600, -1200, -800, -400, and -300, while the reporter activities reached around 100% for the deletion constructs of -200, -100, and -0, indicating that BA-induced reporter activity suppression was restored in the deletion constructs of -200, -100, and -0, and the BA-responsive transcriptional element is located in the range of -300~0 on the ERβ promoter (see **Figure 2E**). We also evaluated the potential epigenetic changes in the range of -300~0 on the ERβ promoter by ChIP analysis. The results showed that there was no significant difference in any of the different treatments on either histone H3 (K9, K14, K18, K23, K27) or H4 (K5, K8, K12, K16) acetylation (see **Figure S4A**). Furthermore, we found that betulinic acid had no effect on histone H4 methylation, including H4K20me1, H4K20me3, and H4K3me1 (see **Figure S4B**). Finally, we evaluated the potential effect of different treatments on H3 methylation. The results showed that there was no effect on the methylation of H3K9me3 and H3K27me2, while 12Z/CTL treatment decreased H3K9me2 and H3K27me3 to 43 and 51%, respectively, compared to the HEEC/CTL group; ERβ knockdown (12Z/shERβ) showed no effect, while BA treatment (12Z/BA) completely reversed 12Z-mediated decreased H3 methylation on the ERβ promoter (see **Figure 2F**). Our results indicate that betulinic acid suppresses the ERβ signaling pathway by modulation of epigenetic changes on the ERβ promoter and subsequent expression.

### Betulinic Acid Mimics ERβ Knockdown-Induced Oxidative Stress in Endometriotic Cells

**Figure 3:**
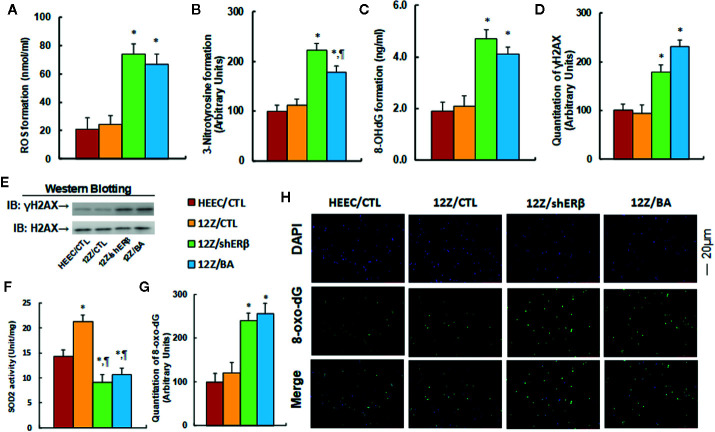
Betulinic acid mimics ERβ knockdown-induced oxidative stress in endometriotic cells. The conditional immortalized HEEC or 12Z cells were either treated by 20 µM betulinic acid (BA) for 24 h or infected by either empty control (CTL) or ERβ knockdown (shERβ) lentivirus; the cells were then harvested for analysis of oxidative stress. **(A)** ROS formation, n = 5. **(B)** Quantitation of 3-nitrotyrosine formation, n = 5. **(C)** 8-OHdG formation, n = 5. **(D)** Quantitation of γH2AX formation. **(E)** Representative γH2AX western blotting band for **(D)**, n = 5. **(F)** SOD2 activity, n = 5. **(G)** Quantitation of 8-oxo-dG formation, n = 5. **(H)** Representative pictures of 8-oxo-dG staining for oxidative stress (green) and DAPI staining for nuclei (blue), n = 4. *P < 0.05, *vs* HEEC/CTL group; ^¶^ *P* < 0.05, *vs* 12Z/CTL group. Data were expressed as mean ± SEM.

We evaluated the effect of ERβ knockdown and betulinic acid-induced oxidative stress in endometriotic cells. The conditional immortalized HEEC or 12Z cells were either treated by 20 µM betulinic acid (BA) for 24 h or infected by either an empty control (CTL) or ERβ knockdown (shERβ) lentivirus, and the cells were then harvested for analysis. Our results showed that ERβ knockdown (12Z/shERβ) and BA treatment (12Z/BA) increased ROS formation to 352 and 319%, respectively (see **Figure 3A**), and increased 3-nitrotyrosine formation to 223 and 178%, respectively (see **Figure 3B**), compared to the HEEC/CTL group; while the oxidative stress showed no significant difference between HEEC and 12Z cells. We then investigated the effect of stimulus TNFα on oxidative stress in those cells (6). The results showed that 12Z cells had significant higher levels of ROS formation (see **Figure S5A**), 3-nitrotyrosin formation (see **Figure S5B**), and 8-OHdG formation (see **Figure S5C**) in 12Z cells compared to HEEC cells in the presence of TNFα. We next measured the effect of betulinic acid on DNA damage. The results showed that treatments of 12Z/shERβ and 12Z/BA increased 8-OHdG formation to 247 and 216%, respectively (see **Figure 3C**) and increased γH2AX formation to 178 and 231%, respectively (see **Figures 3D, E**, and **S6**). Next, we measured SOD2 activity, and the results showed that 12Z/CTL group increased SOD2 activity to 149%, while treatments of 12Z/shERβ and 12Z/BA decreased SOD2 activity to 64 and 74%, respectively, compared to the HEEC/CTL group (see **Figure 3F**). Finally, we evaluated the 8-oxo-dG formation using immunostaining quantitation. The results showed that treatments of 12Z/shERβ and 12Z/BA increased 8-oxo-dG formation to 241 and 256%, respectively, compared to the HEEC/CTL group, while the 12Z/CTL group showed no difference (see **Figures 3G, H**). Our results indicate that betulinic acid mimics ERβ knockdown-induced oxidative stress in endometriotic cells.

### Betulinic Acid Mimics ERβ Knockdown-Induced Mitochondrial Dysfunction and Apoptosis in Endometriotic Cells

**Figure 4:**
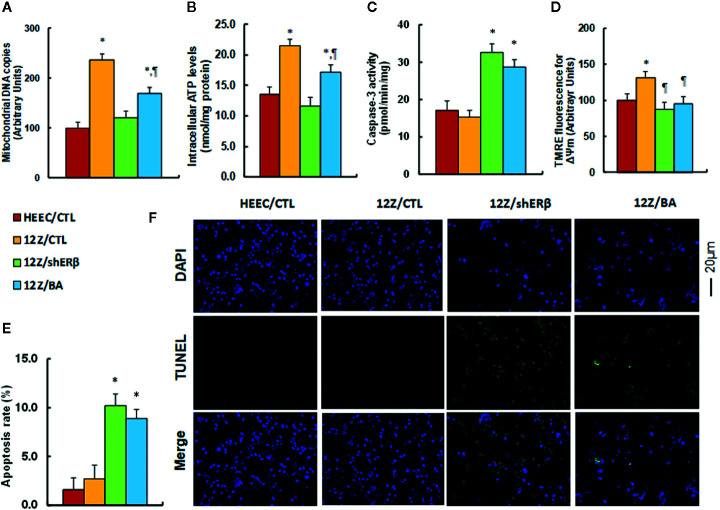
Betulinic acid mimics ERβ knockdown-induced mitochondrial dysfunction and apoptosis in endometriotic cells. The conditional immortalized HEEC or 12Z cells were infected by lentivirus with either empty control (CTL), or ERβ knockdown (shERβ), or treated by 20 µM betulinic acid (BA) for 24 h; the cells were then harvested for analysis of mitochondrial function. **(A)** Mitochondrial DNA copies, n = 4; **(B)** Intracellular ATP levels, n = 5. **(C)** Caspase-3 activity, n = 5. **(D)** Δψm by TMRE fluorescence, n = 5. **(E)** Apoptosis rate by TUNEL assay, n = 5. **(F)** Representative pictures for **(E)**. *P < 0.05, *vs* HEEC/CTL group; ^¶^ *P* < 0.05, *vs* 12Z/CTL group. Data were expressed as mean ± SEM.

We evaluated the effect of ERβ knockdown and betulinic acid-induced mitochondrial dysfunction in endometriotic cells. The results showed that mitochondrial DNA copies (see **Figure 4A**) and intracellular ATP levels (see **Figure 4B**) in the 12Z/CTL group were significantly increased to 236 and 169%, respectively, compared to the HEEC/CTL group. ERβ knockdown (12Z/shERβ) completely normalized this effect, while betulinic acid treatment (12Z/BA) partly normalized this effect. We measured the caspase-3 activity in different treatments. The results showed that 12Z/CTL had no effect, while 12Z/shERβ group increased caspase-3 activity to 191% compared to the HEEC/CTL group, and 12Z/BA mimicked the effect of 12Z/shERβ (see **Figure 4C**). Additionally, mitochondrial membrane potential (ΔΨm) of the 12Z/CTL group significantly increased to 131% compared to the HEEC/CTL group, and treatments of 12Z/shERβ and 12Z/BA completely normalized this effect (see **Figure 4D**). Finally, we evaluated the apoptosis rate. The results showed that 12Z/CTL had no effect, while apoptosis rate in the 12Z/shERβ group increased to 638% compared to the HEEC/CTL group, and 12Z/BA mimicked the effect of 12Z/shERβ (see **Figures 4E, F** ). In addition, the caspase-3 activity and apoptosis rate showed no significant difference between HEEC and 12Z cells. We then investigated the effect of stimulus TNFα in those cells (6). The results showed that 12Z cells had significant lower levels of caspase-3 activity (see **Figure S7A**) and apoptosis rate (see **Figure S7B**) in 12Z cells compared to HEEC cells in the presence of TNFα. The results suggest that betulinic acid mimics ERβ knockdown-induced mitochondrial dysfunction and apoptosis in endometriotic cells.

### ERβ Knockdown Reduces Endometriosis-Mediated Elevated Proinflammatory Cytokine Secretion, and Betulinic Acid Treatment Mimics This Effect

**Figure 5:**
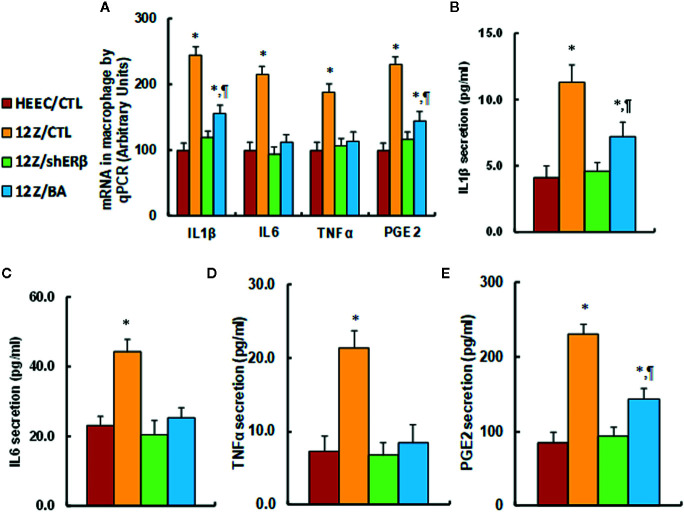
ERβ knockdown reduces endometriosis-mediated elevated proinflammatory cytokine secretion, and betulinic acid treatment mimics this effect. The conditionally immortalized HEEC or 12Z cells were either treated with 20µM betulinic acid (BA) for 24 hours or infected by either empty control (CTL) or ERβ knockdown (shERβ) lentivirus; the cells were then harvested for analysis of proinflammtory cytokine secretion. **(A)** mRNA levels by qPCR, n=4. **(B)** IL1β secretion, n=5. **(C)** IL6 secretion, n=5. **(D)** TNFα secretion, n=5. **(E)** PGE2 secretion, n=5. *P < 0.05, vs HEEC/CTL group; ^¶^*P* < 0.05, vs 12Z/CTL group. Data were expressed as mean ± SEM.

We evaluated the potential effect of ERβ knockdown and betulinic acid-mediated proinflammatory cytokine release in endometriotic cells. We first evaluated the mRNA levels of proinflammatory cytokines, and the results showed that 12Z/CTL group significantly increased mRNA levels of IL1β, IL6, TNFα, and PGE2 to 245, 215, 189, and 231%, respectively, compared to the HEEC/CTL group, while 12Z/shERβ treatment completely normalized this effect, and 12Z/BA treatment completely normalized the mRNA levels of IL6 and TNFα, but partly normalized mRNA levels of IL1β and PGE2 (see **Figure 5A**). Furthermore, we measured cytokine release from cell culture supernatants for IL1β (see **Figure 5B**), IL6 (see **Figure 5C**), TNFα (see **Figure 5D**), and PGE2 (see **Figure 5E**), and an expression pattern similar to that of the mRNA was observed. Our results indicate that ERβ knockdown reduces endometriosis-mediated elevated proinflammatory cytokine secretion, and betulinic acid treatment mimics this effect.

### ERβ Knockdown Reduces Endometriosis-Mediated Cell Proliferation, and Betulinic Acid Treatment Mimics This Effect

**Figure 6:**
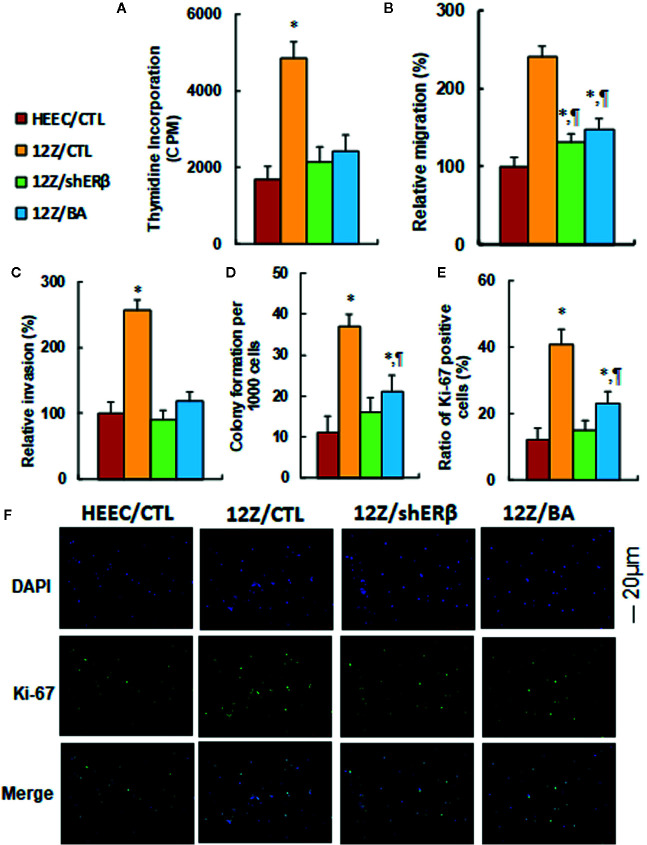
ERβ knockdown reduces endometriosis-mediated cell proliferation, and betulinic acid treatment mimics this effect. The conditional immortalized HEEC or 12Z cells were either treated with 20 µM betulinic acid (BA) for 24 h or infected by either empty control (CTL) or ERβ knockdown (shERβ) lentivirus; the cells were then harvested for analysis of cell proliferation. **(A)** Cell proliferation analysis by thymidine incorporation, n = 5. **(B)** Cell migration assay, n = 5. **(C)** Cell invasion assay, n = 5. **(D)** Colony formation assay in soft agar, n = 5. **(E)** Quantitation of Ki-67 positive cells, n = 3. **(F)** Representative picture for Ki-67 staining. *P < 0.05, *vs* HEEC/CTL group; ^¶^ *P* < 0.05, *vs* 12Z/CTL group. Data were expressed as mean ± SEM.

We evaluated the potential effect of ERβ knockdown and betulinic acid-mediated cell proliferation in endometriotic cells. First, cell proliferation was measured by thymidine incorporation. The results showed that 12Z/CTL group significantly increased thymidine incorporation to 290% compared to the HEEC/CTL group, and treatments of 12Z/shERβ and 12Z/BA completely normalized this effect (see **Figure 6A**). We then evaluated cell migration (see **Figure 6B**) and invasion (see **Figure 6C**). The results showed that the 12Z/CTL group significantly increased cell migration and cell invasion to 241 and 257%, respectively, compared to the HEEC/CTL group, and treatments of 12Z/shERβ and 12Z/BA partly normalized the effect on cell migration, but completely normalized the effect on cell invasion. We also evaluated cell colony formation (see **Figure 6D**) and the ratio of Ki-67 positive cells (see **Figures 6E, F**). The results showed that cell colony formation and the ratio of Ki-67 positive cells in the12Z/CTL group significantly increased to 336 and 342%, respectively, compared to the HEEC/CTL group, and 12Z/shERβ treatment completely normalized this effect, while 12Z/BA treatment partly normalized this effect. Our results indicate that ERβ knockdown reduces endometriosis-mediated cell proliferation, and betulinic acid treatment mimics this effect.

#### Gain of ERβ in Endometrial Cells Promotes Cell Proliferation, While BA Treatment Diminishes This Effect

**Figure 7:**
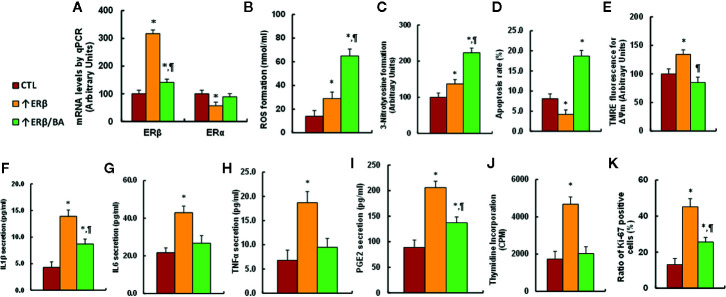
Gain of ERβ in endometrial cells promotes cell proliferation, while BA treatment diminishes this effect. The conditional immortalized HEEC cells were infected by either empty control (CTL) or ERβ expression (↑ERβ) lentivirus, or ERβ expression (↑ERβ) lentivirus plus 20 µM betulinic acid (BA) (↑ERβ/BA) for 24 h, and the cells were harvested for biomedical analysis. **(A)** mRNA levels by qPCR, n = 4. **(B)** ROS formation, n = 5. **(C)** Quantitation of 3-nitrotyrosine formation, n = 5. **(D)** Apoptosis rate by TUNEL assay, n = 5. **(E)** Δψm by TMRE fluorescence, n = 5. **(F)** IL1β secretion, n = 5. **(G)** IL6 secretion, n = 5. **(H)** TNFα secretion, n = 5. **(I)** PGE2 secretion, n = 5. **(J)** Cell proliferation analysis by thymidine incorporation, n = 5. **(K)** Quantitation of Ki-67 positive cells, n = 4. *P < 0.05, *vs* CTL group; ^¶^ *P* < 0.05, *vs* ↑ERβ group. Data were expressed as mean ± SEM.

We investigated the potential effect of ERβ expression and BA treatment in endometrial cells. The conditional immortalized HEEC cells were infected by either empty control (CTL) or ERβ expression (↑ERβ) lentivirus, or ERβ expression (↑ERβ) lentivirus plus 20 µM betulinic acid (BA) (↑ERβ/BA) for 24 h, then the cells were harvested for analysis. We first evaluated the ERβ/ERα mRNA level, and the results showed that ERβ expression (↑ERβ) increased ERβ mRNA level to 317% compared to control (CTL) group, while BA treatment (↑ERβ/BA) significantly reduced ERβ level to 44% compared to ↑ERβ group; on the other hand, ERβ expression (↑ERβ) treatment decreased ERα mRNA to 57%, and BA treatment (↑ERβ/BA) completely reversed this effect ((see **Figure 7A**). We next measured the oxidative stress. We found that ↑ERβ treatment slightly increased ROS formation (see **Figure 7B**) and 3-nitrotyrosine formation (see **Figure 7C**), while ↑ERβ/BA treatment further significantly potentiated the oxidative stress compared to CTL group. We next measured the mitochondrial function. The results showed that ↑ERβ treatment slightly decreased apoptosis rate (see **Figure 7D**) and increased mitochondria membrane potential (see **Figure 7E**), while ↑ERβ/BA treatment significantly further increased the apoptosis rate, and restored mitochondria membrane potential to normal level compared to CTL group. We also measured the proinflammatory cytokine release. The results showed that ↑ERβ treatment significantly increased cytokine releases, including IL1β (see **Figure 7F**), IL6 (see **Figure 7G**), TNFα (see **Figure 7H**), and PGE2 (see **Figure 7I**), while ↑ERβ/BA treatment completely or partly restored cytokine release to normal levels compared to CTL group. We finally evaluated the cell proliferation. The results showed that ↑ERβ treatment significantly increased thymidine incorporation (see **Figure 7J**) and ratio of Ki-67 positive cells (see **Figure 7K**), while ↑ERβ/BA treatment completely or partly restored this effect to normal levels compared to CTL group. Our results indicate that gain of ERβ in endometrial cells promotes cell proliferation and proinflammatory cytokine release, while BA treatment diminishes this effect through increased oxidative stress and apoptosis.

## Discussion

In this study, we demonstrated that gene expression of ERβ and its target genes are significantly increased in endometriotic cells compared to endometrial cells, and BA treatment suppresses ERβ expression by epigenetic changes on the ERβ promoter in endometriotic cells. In addition, BA treatment increases oxidative stress, induces mitochondrial dysfunction, and decreases proinflammatory cytokine release, subsequently inhibiting endometriosis.

### Effect and Role of ERβ and ERα in Endometriosis

Our results showed that ERβ expression was significantly increased in endometriotic cells as well as ERβ target genes, including SOD2, NRF1, COX2, and MMP1, subsequently contributing to endometriosis (EMS) development (4, 5), while ERα expression was significantly decreased, and the ERβ:ERα ratio in endometriotic 12Z cells is increased to 38:1 compared to endometrial HEEC cells as calculated from data in **Figure 1A**, which is consistent with previous report (4, 5). In addition, increased SOD2 expression results in decreased reactive oxygen species (ROS) formation and minimized oxidative stress and increases the invasiveness of cell growth (37), while increased NRF1 expression triggers mitochondrial replication through Tfam (transcription factor A, mitochondrial) (7, 38), providing stronger respiration and metabolic energy for cell proliferation (39). On the other hand, increased COX2 expression involves with inflammation and COX2-derived prostaglandin E2 (PGE2) biosynthesis contributes to EMS-related pain and infertility (1, 13), while increased MMP1 expression is involved with tissue degradation, menstrual bleeding, and invasion of seeded endometriotic explants (14, 15). In addition, gain of ERβ in normal endometrial cells significantly increased cell proliferation, proinflammatory cytokine release together with decreased apoptosis (6). We conclude that increased ERβ expression may be the major driving force for EMS development, and ERβ is the potential therapeutic target for clinical treatment of endometriosis (40).

### Potential Effect of Betulinic Acid on ERβ Expression

Our results showed that betulinic acid (BA) treatment significantly suppresses the expression of ERβ and its target genes through epigenetic changes on the ERβ promoter, subsequently suppressing cellular proliferation and growth. The BA-mediated epigenetic modifications may be transferred into daughter cells during cell proliferation, making the BA-mediated ERβ suppression effect stable and long-lasting. In addition, it has been reported that BA is widely available from common natural sources with relative non-toxicity, making BA a potential novel candidate for drug development for rescuing the epigenetic changes (29).

### Potential Effect of Betulinic Acid on Endometriosis Growth

Our results showed that betulinic acid can significantly inhibit the endometriotic cell growth through specific suppression of ERβ and its target genes in endometriotic cells (28). Betulinic acid treatment significantly increases oxidative stress and DNA damage, inhibits the proinflammatory cytokines release (41), resulting in apoptosis and suppressed cell proliferation in endometriotic cells. Furthermore, the betulinic acid has little effect on the expression of ERα, and it significantly suppresses ERβ expression in endometriotic cells, but has no effect in normal endometrial cells, indicating that betulinic acid can specifically inhibit ERβ expression in endometriosis. Our data showed that betulinic acid may be a potential novel candidate for clinical treatment of endometriosis.

## Conclusions

The endometriotic cells have high expression of ERβ and its target genes, and betulinic acid can specifically suppress ERβ signaling pathway by epigenetic modification on the ERβ promoter, subsequently suppressing endometriosis development. We conclude that betulinic acid inhibits endometriosis through suppression of ERβ signaling pathway.

## Data Availability Statement

The raw data supporting the conclusions of this article will be made available by the authors, without undue reservation.

## Author Contributions

PY wrote the paper. PY, LL, and XL designed, analyzed the data, and interpreted the experiments. YW, ML, MX, and HP performed vector constructions and gene expression analysis. LZ and HuY performed statistical analysis and part of cell proliferation analysis. HeY, HL, and MW performed gene analysis and part of the mapping analysis. DX, MZ, and XC performed the remaining experiments. All authors contributed to the article and approved the submitted version.

## Funding

This study was supported by the Foundation of Situ Yi Renowned Medical Heritage Studio, Scientific Research Project of Wuhan Health and Family Planning Commission #: WZ17A09, Hainan Province Research and Development Key Project #: ZDYF2018214.

## Conflict of Interest

The authors declare that the research was conducted in the absence of any commercial or financial relationships that could be construed as a potential conflict of interest.

## Abbreviations

BA, betulinic acid; ChIP, chromatin immunoprecipitation; COX2, cyclooxygenase 2; EMS, endometriosis; ERβ, estrogen receptor β; IL6, interleukin-6; IL1β, interleukin-1β; MMP1, matrix metalloproteinase-1; NRF1, nuclear respiratory factor-1; PGE2, prostaglandin E2; ROS, reactive oxygen species; SOD2, superoxide dismutase 2; TNFα, tumor necrosis factor-α.
