# Supplementary material for: Betulinic Acid Inhibits Endometriosis Through Suppression of Estrogen Receptor β Signaling Pathway
Source: Front Endocrinol (Lausanne). 2020 Dec 10;11:604648. doi: 10.3389/fendo.2020.604648 (PMC7759155; doi:10.3389/fendo.2020.604648)
Supplement: Supplementary file 1 [file DataSheet_1.docx]

**Betulinic Acid Inhibits Endometriosis through Suppression of Estrogen Receptor β Signaling Pathway**

Dongfang Xiang^1,^*, Min Zhao^2,^*, Xiaofan Cai^3,^*, Yongxia Wang^1^, Lei Zhang^2^, Helen Yao^3^,

Min Liu^1^, Huan Yang^2^, Mingtao Xu^1^, Huilin Li^3^, Huijuan Peng^1^, Min Wang^3^,

Xuefang Liang^1,#^, Ling Li^3,#^, Paul Yao^1,3,#^

**Supplemental Information**

**Data S1.** MATERIALS AND METHODS

**Materials and Reagents.**

Antibodies for β-actin (sc-47778), COX2 (sc-19999), MMP1 (sc-21731), NRF1 (sc-101102), and SOD2 (sc-30080) were obtained from Santa Cruz Biotechnology. Antibodies for acetyl-histone H4 K5, K8, K12, and K16 (H4K5,8,12,16ac, #PA5-40084) were obtained from Invitrogen. Antibodies for anti-histone H3 acetyl K9, K14, K18, K23, K27(H3K9,14,18,23,27ac, ab47915), ERα (ab3575), ERβ (ab3576), H4K20me1 (ab9051), H4K20me3 (ab9053), H4R3me1 (ab17339), H3K9me2 (ab1220), H3K9me3 (ab8898), H3K27me2 (ab24684) and H3K27me3 (ab6002), H2AX (ab20669) and γH2AX (ab2893) were obtained from Abcam. The antibody for 8-oxo-dG (4354-MC-050) was obtained from Novus Biologicals. The 3-nitrotyrosine (3-NT) was measured using 3-Nitrotyrosine ELISA Kit (ab116691 from Abcam) per manufacturers’ instructions. The mitochondrial fraction was isolated using a Pierce Mitochondria Isolation Kit (Pierce Biotechnology) according to manufacturers’ instructions. The protein concentration was measured using the Coomassie Protein Assay Kit (Pierce Biotechnology) per manufacturers’ instructions. Luciferase activity assay was carried out using the Dual-Luciferase™ Assay System (Promega) and the transfection efficiency was normalized using a cotransfected renilla plasmid (1). 17β-estradiol (E2, #E2758) and TNFα (#T0157) were obtained from Sigma.

**Human cell lines.** Human Endometrial Epithelial Cells HEEC (#ABC-TC4601) and Immortalized Human Endometriotic Epithelial Cell Line 12Z (#ABI-TC278D) were obtained from ACCEGEN Biotechnology. The human primary endometrial epithelial cells (EM) and primary endometriotic epithelial cells (EMT) were a kind gift from Dr. Haimou Zhang (from Hubei University) (2). In some of the experiments, the cells for HEEC, EM and EMT were conditionally immortalized using a hTERT lentivirus vector with an extended life span to achieve higher transfection efficiency and experimental stability (3, 4). The cells were maintained in DMEM/F12 medium supplemented with 10% FBS, 1% penicillin and streptomycin and 1% sodium pyruvate at 37°C with 5% CO2.

**Construction of human reporter plasmid**. In order to construct ERβ reporter plasmids, the gene promoters (2kb upstream of the transcription start site plus first exon) were amplified from Ensembl ID: ESR2-201 ENST00000267525.10 by PCR from human genomic DNA and subcloned into the pGL3-basic vector (#E1751, Promega) using restriction sites of Mlu I and Hind III with the following primers: ERβ forward: 5’-gcgc-acgcgt- att tca aga cga gcc tgg cca -3’ (Mlu I) and ERβ reverse: 5’- gtac- aagctt- ctg ttt aca ggt aag gtg tgt -3’ (Hind III). To map promoter activity, the related deletion promoter constructs were generated by PCR methods and subcloned into the pGL3-basic vector (1).

**Generation of human ERβ expression lentivirus***.* The human cDNA for ERβ was obtained from Open Biosystems. The cDNA for human ERβ was subcloned into the pLVX-Puro vector (from Clontech) with the restriction sites of Xho1 and Xba1 using the below primers: ERβ forward primer: 5’- gtac - ctcgag- atg gat ata aaa aac tca cca -3’ (Xho1) and ERβ reverse primer: 5’- gtac - tctaga- tca ctg ctc cat cgt tgc ttc -3’ (Xba1). The ERβ or empty control (CTL) was expressed through Lenti-X™ Lentiviral Expression Systems (from Clontech) per manufacturers’ instructions (5).

**Preparation of ERβ knockdown (shERβ)**. The shRNA lentivirus plasmids for human ERβ (sc-35325-SH) or non-target control (sc-108060) were purchased from Santa Cruz Biotechnology. The related lentiviruses for ERβ and empty control (CTL) were expressed through Lenti-X™ Lentiviral Expression Systems (from Clontech) per manufacturers’ instructions. The purified and condensed lentiviruses were used for in vitro gene knockdown. The knockdown efficiency was confirmed by more than 65% of mRNA reduction compared to the control group in cells using real time PCR (see Table 1).

**RT reaction and real-time quantitative PCR.** Total RNA from treated cells was extracted using the RNeasy Micro Kit (Qiagen), and the RNA was reverse transcribed using an Omniscript RT kit (Qiagen). All the primers were designed using Primer 3 Plus software with the Tm at 60°C, primer size of 21bp, and the product length in the range of 140-160bp (see Table 1). The primers were validated with an amplification efficiency in the range of 1.9-2.1, and the amplified products were confirmed with agarose gel. The real-time quantitative PCR was run on iCycler iQ (Bio-Rad) with the Quantitect SYBR green PCR kit (Qiagen). PCR was performed by denaturing at 95°C for 8 min followed by 45 cycles of denaturation at 95°C, annealing at 60°C, and extension at 72°C for 10s, respectively. 1 µl of each cDNA was used to measure target genes. The β-actin was used as the housekeeping gene for transcript normalization, and the mean values were used to calculate relative transcript levels with the ^ΔΔ^CT method per instructions from Qiagen. In brief, the amplified transcripts were quantified by the comparative threshold cycle method using β-actin as a normalizer. Fold changes in gene mRNA expression were calculated as 2^−ΔΔCT^ with CT = threshold cycle, ΔCT=CT (target gene)-CT(β-actin), and the ΔΔCT =ΔCT (experimental)-ΔCT (reference) (1, 5).

**Western Blotting.** Cells were lysed in an ice-cold lysis buffer (0.137M NaCl, 2mM EDTA, 10% glycerol, 1% NP-40, 20mM Tris base, pH 8.0) with protease inhibitor cocktail (Sigma). The proteins were separated in 10% SDS-PAGE and further transferred to the PVDF membrane. The membrane was incubated with appropriate antibodies, washed and incubated with HRP-labeled secondary antibodies, and then the blots were visualized using the ECL+plus Western Blotting Detection System (Amersham). The blots were quantitated by IMAGEQUANT, and final results were normalized by β-actin (1, 5).

**Luciferase reporter assay.** 1.0×10^5^ of cells were seeded in a 6-well plate with complete medium to grow until they reached 80% confluence. Cells were then cotransfected using 3µg of reporter constructs, together with 0.2µg of pRL-CMV-Luc *Renilla* plasmid (from Promega). After treatment, the cells were harvested and the luciferase activity assays were carried out using the Dual-Luciferase^TM^ Assay System (Promega), and the transfection efficiencies were normalized using a cotransfected *Renilla* plasmid according to manufacturers’ instructions. The reporter activity from different treatments was calculated (1).

**Chromatin Immunoprecipitation (ChIP).** Cells were washed and crosslinked using 1% formaldehyde for 20 min and terminated by 0.1M glycine. Cell lysates were sonicated and centrifuged. 500µg of protein were pre-cleared by BSA/salmon sperm DNA with preimmune IgG and a slurry of Protein A Agarose beads. Immunoprecipitations were performed with the indicated antibodies, BSA/salmon sperm DNA and a 50% slurry of Protein A agarose beads. Input and immunoprecipitates were washed and eluted, then incubated with 0.2mg/ml Proteinase K for 2h at 42˚C, followed by 6h at 65˚C to reverse the formaldehyde crosslinking. DNA fragments were recovered by phenol/chloroform extraction and ethanol precipitation. A ~150bp fragment on the promoter was amplified by real-time PCR (qPCR) using the primers provided in Table 1 (1, 5).

**SOD2 activity assay.** SOD2 was obtained from the mitochondrial fraction that was isolated using a Pierce Mitochondria Isolation Kit (Pierce) according to manufacturers’ instructions. SOD activity was measured as described previously (6). In brief, a stable O2.- source was generated through the conversion action of XOD (xanthine oxidase) from xanthine and was mixed with chemiluminescent (CL) reagents to achieve a stable light emission. The SOD2 sample injection can scavenge O2.- and the subsequent decrease of chemiluminescent response is proportional to the SOD2 activity. This system can have a detection limit of 0.001U.ml-1 with the linear range of 0.03~2.00U.ml-1. The results were normalized by protein concentration and expressed as Units/mg proteins (U/mg) (7).

**Measurement of ROS generation.** Treated cells were seeded in a 24-well plate and incubated with 10μM CM-H2DCFDA (Invitrogen) for 45 min at 37°C, and then the intracellular formation of reactive oxygen species (ROS) was measured at excitation/emission wavelengths of 485/530nm using a FLx800 microplate fluorescence reader (Bio-Tek). The data was normalized as arbitrary units (1, 8).

Measurement of DNA breaks. Comet assay was measured using a CometAssay™ kit (Cat No. TA800) from R&D Systems Inc, and the 8-OHdG formation was measured using an OxiSelect™ Oxidative DNA Damage ELISA Kit (Cat No. STA320, from Cell Biolabs Inc.) according to manufacturers’ instructions. The formation of γH2AX was measured from nuclear extracts by western blotting using H2AX as the input control.

**Evaluation of mitochondrial function.**

*Mitochondrial DNA copies.* The genomic DNA was extracted from cells using a QIAamp DNA Mini Kit (Qiagen) and the mitochondrial DNA was extracted using the REPLI-g Mitochondrial DNA Kit (Qiagen). The purified DNA was used for the analysis of genomic β-actin (marker of the nuclear gene) and ATP6 (ATP synthase F0 subunit 6, marker of the mitochondrial gene) respectively using the qPCR method mentioned above. The primers for genomic β-actin: forward 5’-acc aca gct gag agg gaa atc -3’ and reverse 5’- att gcc gat agt gat gac ctg-3’. The primers for ATP6: forward 5’- tag ggc ttc ttc ccc ata cat -3’ and reverse 5’- tta gtg aga tgg ggg ttc ctt-3’. The mitochondrial DNA copies were obtained from relative ATP6 copies that were normalized by β-actin copies using the ^ΔΔ^CT method.

*Intracellular ATP levels.* Intracellular ATP levels was determined using the luciferin/luciferase-induced bioluminescence system. An ATP standard curve was generated at concentrations of 10^-12^-10^-3^M, and intracellular ATP levels were calculated and expressed as nmol/mg protein (8).

*Measurement of apoptosis.* Apoptosis was evaluated by TUNEL assay using the In Situ Cell Death Detection Kit™ (Roche). Cells were fixed in 4% paraformaldehyde and labeled using TUNEL reagents. Stained cells were photographed using a fluorescence microscope and further quantified by FACS analysis. Caspase-3 activity was determined by the ApoAlert caspase assay kit (Clontech). Treated cells were harvested and 50 µg of proteins were incubated with the fluorogenic peptide substrate Ac-DEVD-7-amino-4-trifluoromethyl coumarin (AFC). The initial rate of free AFC release was measured using a FLx800 microplate reader (Bio-Tek) at excitation/emission wavelengths of 380/505nm, and enzyme activity was calculated as pmol/min/mg (8).

*Mitochondrial membrane potential (Δψm).* The Δψm was measured by TMRE (from Molecular Probes T-669) staining. A 600μM T-669 stock solution was prepared using DMSO. Cells were grown on coverslips before being immersed in 600nM TMRE for 20min at 37°C to load them with dye. Afterwards, the labeling medium was aspirated and cells were immersed in 150nM TMRE to maintain the equilibrium distribution of the fluorophore. Live cells were mounted onto coverslips and the confocal microscope was used to image the cells using 548nm excitation/573nm emission filters. The intensity of TMRE fluorescence was measured using Image J software. Data from 10-20 cells were collected for each experimental condition and mean values of fluorescence intensity ± SEM were calculated.

**Analysis of cytokines**. Human cytokines, including IL-1β, IL-6, and TNF-α from in vitro cell culture supernatant, were measured using [Human IL-1 beta/IL-1F2 Quantikine ELISA Kit (#DLB50](https://www.rndsystems.com/products/human-il-1-beta-il-1f2-quantikine-elisa-kit_dlb50)), [Human IL-6 Quantikine ELISA Kit (#D6050](https://www.rndsystems.com/products/human-il-6-quantikine-elisa-kit_d6050)) and [Human TNF-alpha Quantikine ELISA Kit](https://www.rndsystems.com/products/human-tnf-alpha-quantikine-elisa-kit_dta00d) (#[DTA00D](https://www.rndsystems.com/products/human-tnf-alpha-quantikine-elisa-kit_dta00d)), respectively; and the PGE2 was measured by [Prostaglandin E2 Parameter Assay Kit](https://www.rndsystems.com/products/prostaglandin-e2-parameter-assay-kit_kge004b) (#[KGE004B](https://www.rndsystems.com/products/prostaglandin-e2-parameter-assay-kit_kge004b)) according to manufacturers’ instructions from R&D Systems (9).

**Immunostaining**. The treated cells were transferred to cover slips, and the cells were fixed in 4% paraformaldehyde for 20 min before being incubated with 0.3% Triton X-100 in PBS for 15 min. After blocking with 5% goat serum in PBS at room temperature for 30 min, cells were incubated with anti-mouse antibody for either 8-oxo-dG (# 4354-MC-050, from Novus Biologicals) or Ki-67 (# sc-101861, from Santa Cruz Biotechnology) for 12 h at 4°C and subsequently with secondary antibody Alexa Fluor 488. The cover slips were then mounted by antifade Mountant with DAPI (staining nuclei, in blue). The photographs were taken using a [Confocal Laser Microscope](https://www.sogou.com/link?url=DSOYnZeCC_qw-OVKG_MsR3KENashJ6PPMhOejy_Q5JJflCntg_rzjU2lo9-QKkufX5Qp7YP6841C08P_Gzn4lQD4cR4JDdkk5sef3Ee0PfoOX3hBKf-DUA..) (Leica, 20x lens) and quantitated by Image J. software.

**DNA synthesis by [^3^H]-thymidine incorporation**. Cell proliferation was evaluated as the rate of DNA synthesis by [^3^H]-methylthymidine incorporation (10). Cells were pooled in 24-well plates until they reached 80% confluence and then the indicated chemicals were added and incubated for 24 hours. At the end of the treatment, cells were incubated with serum-free media containing ^3^H-methylthymidine (0.5 µCi/well) for 2 hours and then washed twice with PBS. Cellular DNA was precipitated using 10% trichloroacetic acid and solubilized with 0.4M NaOH (0.5 ml/well). Incorporation of ^3^H-methylthymidine into the DNA was measured in a scintillation counter and was determined as counts per minute (CPM) (1).

**Colony formation in soft agar**. This assay is a method for evaluating the ability of individual cell lines to grow in an anchorage-independent manner. Cells were resuspended in DMEM containing 5% FBS with 0.3% agarose and layered on top of 0.5% agarose in DMEM on 60-mm plates. 1000 cells were seeded in 60mm soft agar dishes for 30 days. The dishes were examined twice per week, and colonies that grew beyond 50mm in diameter were scored as positive. Each experiment was done in quadruplicate (1).

**Migration and invasion assays**. Cell migration and invasion assays were performed in 24-well chemotaxis plates with an 8μm polycarbonate filter membrane. The plates were coated with 20μg Matrigel for invasion assays and uncoated for migration assays. Invasion and migration were expressed as the number of migrated cells bound per microscopic field and averaged from at least four fields per assay in at least 4 experiments (11, 12).

**Statistical analysis**. The data was given as mean ± SEM, and all the experiments were performed at least in quadruplicate unless otherwise indicated. The unpaired Student’s t-tests or one-way analysis of variance (ANOVA) followed by the Turkey−Kramer test was used to determine statistical significance of different groups by SPSS 22 software, and a *P* value of <0.05 was considered significant.

REFERENCES

1. Zhang H, Li L, Li M, Huang X, Xie W, Xiang W, et al. Combination of betulinic acid and chidamide inhibits acute myeloid leukemia by suppression of the HIF1alpha pathway and generation of reactive oxygen species. *Oncotarget.* 2017;8(55):94743-58.

2. Gou Y, Li X, Li P, Zhang H, Xu T, Wang H, et al. Estrogen receptor beta upregulates CCL2 via NF-kappaB signaling in endometriotic stromal cells and recruits macrophages to promote the pathogenesis of endometriosis. *Hum Reprod.* 2019;34(4):646-58.

3. Bodnar AG, Ouellette M, Frolkis M, Holt SE, Chiu CP, Morin GB, et al. Extension of life-span by introduction of telomerase into normal human cells. *Science.* 1998;279(5349):349-52.

4. Kong D, Zhan Y, Liu Z, Ding T, Li M, Yu H, et al. SIRT1-mediated ERbeta suppression in the endothelium contributes to vascular aging. *Aging Cell.* 2016;15(6):1092-102.

5. Zou Y, Lu Q, Zheng D, Chu Z, Liu Z, Chen H, et al. Prenatal levonorgestrel exposure induces autism-like behavior in offspring through ERbeta suppression in the amygdala. *Mol Autism.* 2017;8:46.

6. Yao D, Vlessidis AG, Gou Y, Zhou X, Zhou Y, and Evmiridis NP. Chemiluminescence detection of superoxide anion release and superoxide dismutase activity: modulation effect of Pulsatilla chinensis. *Anal Bioanal Chem.* 2004;379(1):171-7.

7. Kong D, Zhan Y, Liu Z, Ding T, Li M, Yu H, et al. SIRT1-mediated ERbeta suppression in the endothelium contributes to vascular aging. *Aging Cell.* 2016.

8. Yao D, Shi W, Gou Y, Zhou X, Yee Aw T, Zhou Y, et al. Fatty acid-mediated intracellular iron translocation: a synergistic mechanism of oxidative injury. *Free Radic Biol Med.* 2005;39(10):1385-98.

9. Kobayashi EH, Suzuki T, Funayama R, Nagashima T, Hayashi M, Sekine H, et al. Nrf2 suppresses macrophage inflammatory response by blocking proinflammatory cytokine transcription. *Nat Commun.* 2016;7:11624.

10. Somasundaram K, and El-Deiry WS. Inhibition of p53-mediated transactivation and cell cycle arrest by E1A through its p300/CBP-interacting region. *Oncogene.* 1997;14(9):1047-57.

11. Han HJ, Russo J, Kohwi Y, and Kohwi-Shigematsu T. SATB1 reprogrammes gene expression to promote breast tumour growth and metastasis. *Nature.* 2008;452(7184):187-93.

12. Yu OM, Benitez JA, Plouffe SW, Ryback D, Klein A, Smith J, et al. YAP and MRTF-A, transcriptional co-activators of RhoA-mediated gene expression, are critical for glioblastoma tumorigenicity. *Oncogene.* 2018.

FIGURE S1

**Figure S1. Representative pictures of Western Blotting full blots for Figure 1c.**

FIGURE S2

**Figure S2. Representative pictures of Western Blotting full blots for Figure 2c.**

FIGURE S3

**Figure S3. BA treatment has little effect on the expression of ERα and ERβ in HEEC cells.** HEEC cells were treated by different concentrations of betulinic acid (BA) for 24 hours, then harvested for analysis of ERα and ERβ mRNA by qPCR, n=4. *, *P*<0.05, vs 0μM BA treatment group. Data were expressed as mean ± SEM.

FIGURE S4

**Figure S4. Betulinic acid-mediated epigenetic changes on the ERβ promoter.** The conditional immortalized HEEC or 12Z cells were infected by lentivirus with either empty control (CTL) or ERβ knockdown (shERβ), or treated by 20µM betulinic acid (BA) for 24 hours, then cells were harvested for ChIP analysis. (a) Histone acetylation on ERβ promoter using H3K9,14,18,23,27ac and H4K5,8,12,16ac antibodies, n=4. (b) Histone H4 methylation on ERβ promoter, n=4. Data were expressed as mean ± SEM.

FIGURE S5

**Figure S5. Potential effect of stimulus 50ng/ml of TNFα on oxidative stress.** The conditional immortalized HEEC or 12Z cells were infected by lentivirus with either empty control (CTL) or ERβ knockdown (shERβ), or treated by 20µM betulinic acid (BA) for 24 hours, and the cells were stimulated by 50ng/ml of TNFα for 8 hours before the cells were harvested for analysis of oxidative stress. (a) ROS formation, n=5. (b) Quantitation of 3-nitrotyrosine formation, n=5. (c) 8-OHdG formation, n=5. *, *P*<0.05, vs HEEC/CTL group; ¶, *P*<0.05, vs 12Z/CTL group. Data were expressed as mean ± SEM.

FIGURE S6

**Figure S6. Representative pictures of Western Blotting full blots for Figure 3e**.

FIGURE S7

**Figure S7. Potential effect of stimulus 50ng/ml of TNFα on caspase-3 activity and apoptosis.** The conditional immortalized HEEC or 12Z cells were infected by lentivirus with either empty control (CTL) or ERβ knockdown (shERβ), or treated by 20µM betulinic acid (BA) for 24 hours, and the cells were stimulated by 50ng/ml of TNFα for 8 hours before the cells were harvested for analysis. (a) Caspase-3 activity, n=5. (b) Apoptosis rate by TUNEL assay, n=5. *, *P*<0.05, vs HEEC/CTL group; ¶, *P*<0.05, vs 12Z/CTL group. Data were expressed as mean ± SEM.
